# Primary breast lymphoma in males—a report of two cases with a review of the literature

**DOI:** 10.3332/ecancer.2013.347

**Published:** 2013-09-05

**Authors:** KN Lokesh, Vishwanath Sathyanarayanan, KC Lakshmaiah, TM Suresh, D Lokanatha, K Govinda Babu, Linu Abraham Jacob, Suresh Babu

**Affiliations:** Department of Medical Oncology, Kidwai Memorial Institute of Oncology, Bangalore, Karnataka 560029, India

**Keywords:** breast, diffuse large B cell lymphoma, small lymphocytic lymphoma

## Abstract

Primary breast lymphoma (PBL) in males is a rare clinical entity and has been reported in anecdotal case reports up until now. We report two cases of PBL from a tertiary care centre in Southern India. A 46-year-old male presented with a lump in the right breast with right axillary lymphadenopathy; a biopsy with immunohistochemistry showed neoplastic cells positive for CD 20 and negative for CD 30, epithelial membrane antigen, anaplastic lymphoma kinase, suggestive of diffuse large B cell lymphoma stage IIEA. He received three cycles of CHOP (cyclophosphamide, adriamycin, vincristine, and prednisolone) chemotherapy, then developed a cerebrovascular accident, and died. The other patient was a 60-year-old male with a left breast lump with left axillary lymphadenopathy. The biopsy with immunohistochemistry showed neoplastic cells positive for CD 23 and CD 5, suggestive of small lymphocytic lymphoma stage IIEA. Initially he received three cycles of cyclophosphamide, vincristine, and prednisolone (COP) and defaulted. One year later, he received six cycles of COP chemotherapy, developed progressive disease, and was lost to follow-up. The literature on PBL in males was reviewed. To conclude, PBL in males is an extremely rare disease and can mimic breast cancer. A strong index of suspicion with early diagnosis by biopsy with immunohistochemistry and treatment with rituximab- and anthracycline-based chemotherapy followed by radiotherapy will improve overall survival.

## Introduction

Around 1% of all malignant breast tumours occur in males, and primary breast lymphoma (PBL) in males is an extremely rare clinical entity and can masquerade as breast cancer in its clinical presentation. Early diagnosis and treatment with a combination of radiotherapy and chemotherapy are important.

## Case description

We describe two cases of PBL in males from Kidwai Memorial Institute of Oncology, a tertiary care oncology centre in Southern India.

### Case 1

A 46-year-old male presented with a palpable mass in the right breast of one month duration. He denied B symptoms such as fever, weight loss, or drenching night sweats. On examination, he had an Eastern co-operative oncology group performance status (ECOG PS) of 1.5. The right breast lump was 3 × 4 cm with 1 × 1 cm right axillary lymphadenopathy. The breast lump and axillary lymph node biopsy with immunohistochemistry revealed large B cells positive for CD 20 and negative for CD 30, epithelial membrane antigen (EMA), and anaplastic lymphoma kinase (ALK), suggestive of diffuse large B cell lymphoma (DLBCL) of right breast (Ann-Arbor stage IIEA). He was not able to afford rituximab and received three cycles of chemotherapy with cyclophosphamide 750 mg/m^2^, adriamycin 50 mg/m^2^, vincristine 1.4 mg/m^2^, and prednisolone 100 mg/day for five days (CHOP), following which he developed a cerebrovascular accident with right hemiparesis and died.

### Case 2

A 60-year-old male presented with a palpable mass in the left breast of four months duration. He denied B symptoms. On examination, ECOG PS was 2. He had a 5 × 4 cm palpable mass in the left breast and 2 × 2 cm left axillary lymphadenopathy. Examinations of the heart, lungs, and abdomen were normal. The breast lump and axillary lymph node biopsy with immunohistochemistry revealed neoplastic cells positive for CD 23 and CD 5, suggestive of a small lymphocytic lymphoma (SLL) of the left breast (Ann-Arbor stage IIEA). He received chemotherapy with cyclophosphamide, vincristine, prednisolone (COP) for two cycles and defaulted for one year. He then received six cycles of COP, following which he developed a progressive disease with bilateral supraclavicular lymphadenopathy. He was scheduled for further second-line chemotherapy but was lost to follow-up.

The clinical profile of our patients and a review of the literature have been outlined in Table 1.

## Discussion

PBL is a rare neoplasm of the breast with an incidence of less than 0.5% of primary breast malignancies [1]. The incidence of PBL in men is extremely low, with around 25 cases reported so far [2]. PBL is defined as those who had no evidence of disease beyond the breast or ipsilateral axillary lymph nodes as seen in both of our cases [3]. DLBCL is the most frequent histological type, while mucosa-associated lymphoid tissue-type lymphoma and follicular lymphoma are seen rarely. However, SLL of the breast as seen in one of our cases is an extremely rare type of PBL [4]. Ryan, in his study on PBL, concluded that the use of anthracycline-containing chemotherapy and radiotherapy is strongly associated with longer overall survival, especially in early-stage disease [5]. Our patients also received anthracycline-based chemotherapy. One of our patients (46-year-old male with DLBCL) received three cycles of chemotherapy and died of a cerebrovascular accident with hemiparesis, and the other patient with SLL was lost to follow-up after six cycles of chemotherapy with progressive disease. Studies have also shown that mastectomy is not of any benefit [6]. Rituximab added to six cycles of CHOP-like chemotherapy improved long-term outcomes for patients with good-prognosis DLBCL, but our patients with DLBCL could not afford rituximab therapy [7]. To the best of our knowledge and following our literature search, SLL of the breast has not been reported in males; Park *et al* reported a case of SLL in the female breast in his case series of nine patients who were treated with CHOP chemotherapy and radiotherapy [8].

## Conclusion

PBL in males is an extremely rare entity and can mimic breast cancer. A strong index of suspicion with early diagnosis by biopsy with immunohistochemistry and treatment with rituximab-based chemotherapy followed by radiotherapy will improve overall survival.

## Conflicts of interest

The authors have no conflicts of interest to declare.

## Acknowledgments

We would like to thank the staff in the Department of Medical Oncology at Kidwai Memorial Institute of Oncology for their help and support in preparing this manuscript.

## Figures and Tables

**Table 1.** The clinical profile, treatment, and outcome of PBL in males, with a review of the literature.

	Age/sex	Presentation	Histology	Stage	Treatment	Outcome
Mpallas *et al* (2004) [3]	67 years/M	Mass (R) breast + (R) ALN	DLBCL	N.A.	N.A	Died
Miura *et al* (2009) [11]	64 years/M	Mass (L) breast	DLBCL	IEA	R-CHOP six cycles + IFRT 50 Gy	CR EFS – 12 months
Rathod *et al* (2011) [9]	48 years/M	Mass (L) breast	DLBCL	N.A.	CHOP six cycles	PR
Mahmood *et al* (2011) [10]	50 years/M	Mass (L) breast + (L) ALN	DLBCL	IIIA	N.A.	N.A.
Mantia *et al* (2012) [4]	54 years/M	Mass in (L) breast initially, followed by (R) breast mass two years later	FL grade 3	III A	R-CHOP six cycles + lumpectomy	Died of AML
Ko *et al* (2012) [12]	51 years/M	Mass (L) breast	ALK negative ALCL	N.A	CHOP five cycles	One year EFS
Present study (2013)	46 years/M	Mass (R) breast + (R) ALN	DLBCL	II EA	CHOP three cycles	Died
Present study (2013)	60 years/M	Mass (L) breast + (L) ALN	SLL	IIEA	Three cycles of COP and defaulted, six cycles COP one year later	Lost to follow-up

ALK, anaplastic large cell kinase; ALCL, anaplastic large cell lymphoma; AML, acute myeloid leukaemia; ALN, axillary lymphadenoapthy; CR, complete remission; CHOP, cyclophosphamide, H, doxorubicin, O, vincristine, P, prednisolone; DLBCL, diffuse larger B cell lymphoma; EFS, event-free survival; IFRT, involved field radiotherapy; R, CHOP, rituximab, cyclophosphamide, doxorubicin, vincristine, prednisolone; FL, follicular lymphoma; L, left; M, male; N.A, not available; R, right.
